# The role of speckle tracking echocardiography for prognostication in patients with severe sepsis or septic shock

**DOI:** 10.1186/s13054-016-1451-x

**Published:** 2016-09-12

**Authors:** Filippo Sanfilippo, Cristina Santonocito, Giovanna Panarello, Antonio Arcadipane

**Affiliations:** Department of Anesthesia and Intensive Care, IRCCS-ISMETT (Istituto Mediterraneo per i Trapianti e Terapie ad alta specializzazione), Via Tricomi 5, 90127 Palermo, Italy

Ng et al. [1] reported the results of a very interesting study on sepsis-induced myocardial dysfunction. The authors elegantly matched patients with septic shock (90-day mortality of 41.9 %) with a control group of patients with sepsis (90-day mortality of 0 %) using a relatively novel and more sophisticated echocardiographic approach for the diagnosis of myocardial dysfunction in septic patients—speckle tracking echocardiography. With this approach, systolic dysfunction was assessed by means of global longitudinal strain (GLS) and showed a greater degree of myocardial dysfunction in patients with septic shock compared with controls (GLS −14.5 versus −18.3 %, respectively). On the other hand, left ventricular ejection fraction (LVEF) was similar among groups, again confirming that LVEF does not seem appropriate for evaluating myocardial dysfunction in such a population. This finding is in agreement with recent evidence suggesting no correlation between LVEF and mortality in patients with severe sepsis or septic shock, while an association between diastolic dysfunction and mortality has been suggested [2].

However, caution should be exercised regarding the interpretation of the results on the reversibility of myocardial dysfunction. Indeed, Ng et al. [1] showed the reversibility of septic cardiomyopathy in patients weaned off from vasopressors (n = 23), as demonstrated by a significant improvement in GLS (from −14.6 to −16.0 %, mean difference −1.4 %). Nonetheless, a similar improvement (from −14.2 to −16.1 %, mean difference −1.9 %) was found also in patients not successfully weaned off from vasopressors, and such similar—though non-significant—improvement may just be the result of a smaller sample (n = 10). For instance, De Geer et al. [3] studied patients with septic shock and found no correlation between GLS and systemic vascular resistance index or vasopressor dosage; more interestingly, the authors found no changes in GLS over the time, performing three serial echocardiographic exams (day 1, days 3–4, and after intensive care discharge). Therefore, the question on the reversibility of myocardial dysfunction remains unanswered and larger studies are needed.

Moreover, the authors failed to report the difference in GLS values at the first echocardiographic assessment between survivors and non-survivors of the septic shock group. Indeed, this is an important aspect of ongoing research, since conflicting results have been published so far; for instance, a significant association between less negative GLS values and mortality has been shown by a relatively large study [4], while other smaller studies have not confirmed such results [3, 5].

## References

[1] Ng PY, Sin WC, Ng AK, Chan WM (2016). Speckle tracking echocardiography in patients with septic shock: a case control study (SPECKSS). Crit Care.

[2] Sanfilippo F, Corredor C, Fletcher N, Landesberg G, Benedetto U, Foex P, Cecconi M. Diastolic dysfunction and mortality in septic patients: a systematic review and meta-analysis. Intensive Care Med. 2015;41(6):1004–13. doi: 10.1007/s00134-015-3748-7. 10.1007/s00134-015-3748-7 25800584

[3] De Geer L, Engvall J, Oscarsson A (2015). Strain echocardiography in septic shock--a comparison with systolic and diastolic function parameters, cardiac biomarkers and outcome. Crit Care..

[4] Chang WT, Lee WH, Lee WT, Chen PS, Su YR, Liu PY, Liu YW, Tsai WC (2015). Left ventricular global longitudinal strain is independently associated with mortality in septic shock patients. Intensive Care Med.

[5] Orde SR, Pulido JN, Masaki M, Gillespie S, Spoon JN, Kane GC, Oh JK (2014). Outcome prediction in sepsis: speckle tracking echocardiography based assessment of myocardial function. Crit Care.

[6] Cheng YC, Chen LM, Chang MH, Chen WK, Tsai FJ, Tsai CH, Lai TY, Kuo WW, Huang CY, Liu CJ (2009). Lipopolysaccharide upregulates uPA, MMP-2 and MMP-9 via ERK1/2 signaling in H9c2 cardiomyoblast cells. Mol Cell Biochem.

