# Supplementary material for: The role of speckle tracking echocardiography for prognostication in patients with severe sepsis or septic shock
Source: Crit Care. 2016 Sep 12;20(1):284. doi: 10.1186/s13054-016-1451-x (PMC5018936; doi:10.1186/s13054-016-1451-x)
Supplement: Related texts

# Reply: Author’s response

**Authors:** Pauline Yeung Ng, Wai Ching Sin, Andrew Kei-yan Ng

We thank Dr Sanfilippo et al. for their interest and comments on our publication about the use of speckle tracking echocardiography in sepsis [1].

They rightly pointed out that the reversibility of septic cardiomyopathy in patients weaned off from vasopressors, and the absence of such in patients not successfully weaned off, may be confounded by a small sample size. In a similar study by De Geer et al. [3], although serial echocardiographic exams did not show a significant reversibility in global longitudinal strain (GLS), their data almost reached statistical significance (*P* = 0.10) and, more importantly, displayed a similar pattern of initial impairment and subsequent improvement upon follow-up. It would be interesting if the authors can present data from subgroup analyses of survivors and non-survivors.

In order to accurately study the reversibility of septic cardiomyopathy, the ideal timing of the reassessment echocardiographic examination needs to be defined. Laboratory studies have shown that lipopolysaccharides in sepsis can induce cardiac fibrosis [6], which may or may not produce lasting effects on myocardial contractility. We chose the time of weaning off vasopressors to look at early recovery measurable during the patients’ stay in intensive care. But for the full extent of myocardial impairment/recovery to be appreciated, the follow-up echocardiographic examination may be more suitably performed upon patients’ full recovery and discharge from hospital.

Finally, larger studies are required to establish the relationship between GLS at admission and mortality. In our study, our data may be affected by relatively low 30-day mortality (18.2 %) but high 90-day mortality (41.9 %). Taking into consideration a mean ICU stay of 12.5 days, most of the mortality occurred outside the ICU, where the cause of death may not be related to sepsis.

We believe that speckle tracking echocardiography represents an exciting tool in septic cardiomyopathy and septic shock. Further large well-designed studies will help to clarify current conflicting results and unanswered questions.

## Abbreviations

GLS, Global longitudinal strain; LVEF, Left ventricular ejection fraction.
